# Cost-Effectiveness of Magnetic Resonance Imaging with a New Contrast Agent for the Early Diagnosis of Alzheimer's Disease

**DOI:** 10.1371/journal.pone.0035559

**Published:** 2012-04-20

**Authors:** Maria Biasutti, Natacha Dufour, Clotilde Ferroud, William Dab, Laura Temime

**Affiliations:** 1 Laboratoire Modélisation et Surveillance des risques sanitaires – Conservatoire national des Arts et Métiers – 75003, Paris, France; 2 Ecole des Ponts ParisTech – 77700, Marne la Vallée, France; 3 Laboratoire Transformations Chimiques et Pharmaceutiques – Conservatoire national des Arts et Métiers – 75003, Paris, France; Johns Hopkins Bloomberg School of Public Health, United States of America

## Abstract

**Background:**

Used as contrast agents for brain magnetic resonance imaging (MRI), markers for beta-amyloid deposits might allow early diagnosis of Alzheimer's disease (AD). We evaluated the cost-effectiveness of such a diagnostic test, MRI+CLP (contrastophore-linker-pharmacophore), should it become clinically available.

**Methodology/Principal Findings:**

We compared the cost-effectiveness of MRI+CLP to that of standard diagnosis using currently available cognition tests and of standard MRI, and investigated the impact of a hypothetical treatment efficient in early AD. The primary analysis was based on the current French context for 70-year-old patients with Mild Cognitive Impairment (MCI). In alternative “screen and treat” scenarios, we analyzed the consequences of systematic screenings of over-60 individuals (either population-wide or restricted to the ApoE4 genotype population). We used a Markov model of AD progression; model parameters, as well as incurred costs and quality-of-life weights in France were taken from the literature. We performed univariate and probabilistic multivariate sensitivity analyses.

The base-case preferred strategy was the standard MRI diagnosis strategy. In the primary analysis however, MRI+CLP could become the preferred strategy under a wide array of scenarios involving lower cost and/or higher sensitivity or specificity. By contrast, in the “screen and treat” analyses, the probability of MRI+CLP becoming the preferred strategy remained lower than 5%.

**Conclusions/Significance:**

It is thought that anti-beta-amyloid compounds might halt the development of dementia in early stage patients. This study suggests that, even should such treatments become available, systematically screening the over-60 population for AD would only become cost-effective with highly specific tests able to diagnose early stages of the disease. However, offering a new diagnostic test based on beta-amyloid markers to elderly patients with MCI might prove cost-effective.

## Introduction

Alzheimer's disease (AD) is the main cause of dementia in older people, with approximately 26 million cases worldwide [1], [2], [3]. What is more, a major increase in this prevalence is expected in years to come [3]. In France, while 850.000 people were diagnosed with AD in 2004, 2.1 million may be affected by 2040 [4].

There are currently no treatments that may cure AD or halt the course of the disease. However, in recent years, drugs such as acetylcholinesterase inhibitors have demonstrated efficacy at reducing the intensity of certain symptoms. Moreover, new avenues for research are being investigated. Many scientists believe that one of the main causes of the AD has to do with beta-amyloid, microscopic protein which accumulate throughout the cortex of Alzheimer patients. This is called the “amyloid hypothesis” [2]. They believe that the destruction of brain cells seen in AD is caused by defects in the way beta-amyloid is produced, how it accumulates and how it is eliminated. Animal studies in mice have suggested that anti-beta-amyloid drugs can reduce brain amyloid level and improve memory problems in diseases similar to AD. At the present time, there is no clear evidence that these drugs can improve Alzheimer symptoms or protect brain cells but it is thought that they could halt the development of dementia in patients with early stage AD [5].

With these prospects of further therapeutic developments, attention has now focused on improving the sensitivity and specificity of diagnostic tools, and in developing tools that would allow early diagnosis.

The current standard diagnostic strategy of AD generally comprises a detailed history, a standardized assessment of cognition and functional status and laboratory testing. Brain imaging examinations such as nonenhanced computed tomography imaging, positron emission tomography imaging, or magnetic resonance imaging (MRI) are also sometimes used in order to exclude other conditions or to measure brain atrophy.

Finding more accurate diagnostic tools implies discovering non-invasive sensitive and specific biomarkers for AD. One avenue of research lies in the detection of β-amyloid plaques [6], [7]. This detection could be achieved through the use of new contrast agents for MRI which bind to β-amyloid plaques, thus allowing a valid diagnosis of AD at a very early stage [6], [8].

A few studies have attempted to assess the cost-effectiveness of imaging diagnosis tools for AD [9], [10], [11], or to evaluate the impact of screening the general population [12]. However, none has focused on combinations of MRI and new contrast agents.

In this study, we evaluate the cost-effectiveness of a diagnostic strategy based on MRI with a new contrast agent detecting β-amyloid plaques (contrastophore-linker-pharmacophore or CLP). We compare this strategy with other current standard diagnostic strategies. In the primary analysis, the new strategy is simply introduced as an alternative to current diagnostic tools and made available to the same population. In this setting, we investigate the consequences of the introduction of a new AD treatment with significant efficacy at an early stage of the disease.

In alternative scenarios, we assume that the availability of this new treatment would naturally raise the issue of the opportunity of screening for AD. We thus evaluate the cost-effectiveness of diagnostic strategies in the context of hypothetical national screening programs.

## Methods

### 1. Framework of the cost-effectiveness analyses

We performed cost-effectiveness analyses in order to compare the costs and benefits of several alternative diagnostic strategies in the French context. Costs were measured in Euros (€) and benefits were measured in quality-adjusted life-years (QALYs), which assign to each year of life a weight between 0 (dead) and 1 (perfect health).

A strategy was considered dominated if another strategy had a better or similar efficacy at a lower cost; conversely, a strategy was considered strongly dominant when it was both more effective and cheaper than all other strategies. We computed incremental cost-effectiveness ratios (ICERs), in which changes in resource use, compared with the next best strategy, were included in the numerator, while additional health effects, compared with the next best strategy, were included in the denominator. Finally, we compared ICERs to the willingness-to-pay (WTP) for an additional QALY, which was assumed equal to three times the gross national product per capita in France, as recommended by the World Health Organization Choice working group [13], [14], that is, 76.171€ per QALY in 2009. If no strategy was strongly dominant, the preferred strategy was that with the highest ICER under the willingness-to-pay threshold.

The study was conducted from a societal perspective, meaning it included all costs and benefits, no matter who incurred them. Future costs and QALYs were discounted at 5% annually.

The cost-effectiveness analyses were conducted using the TreeAge software [15]. We performed expected value analyses, based on the computation over simulated cycles of percentages of a hypothetical cohort in each modeled AD stage. For the multivariate sensitivity analyses, we also performed Monte Carlo simulations with 10.000 trials, in order to derive the distribution of incremental cost-effectiveness ratios for the MRI+CLP strategies, as well as acceptability curves for all strategies.

Table 1 details the compared strategies.

**Table 1:** Compared strategies.

Strategy	Tests	Imaging
Standard diagnosis	Laboratory tests, clinical examination, cognition test	None
Standard MRI	Laboratory tests, clinical examination, cognition test	Non-enhanced MRI
MRI+CLP	Laboratory tests, clinical examination, cognition test	MRI with a new contrast agent detecting β-amyloid plaques

#### 1.1. Primary analysis

In the primary analysis, three diagnostic strategies were compared over a three year period for a cohort of 70 year-old individuals consulting for the first time following mild cognitive impairment (MCI) symptoms:

the standard diagnosis strategy based on an interview with an AD specialist, cognition tests such as mini-mental state evaluation (MMSE) and laboratory tests (standard diagnosis strategy)
a strategy combining the standard strategy with standard MRI (standard MRI strategy)
and a strategy combining the standard strategy with MRI used with a new contrast agent detecting β-amyloid plaques (MRI+CLP strategy)

**Figure 1:**
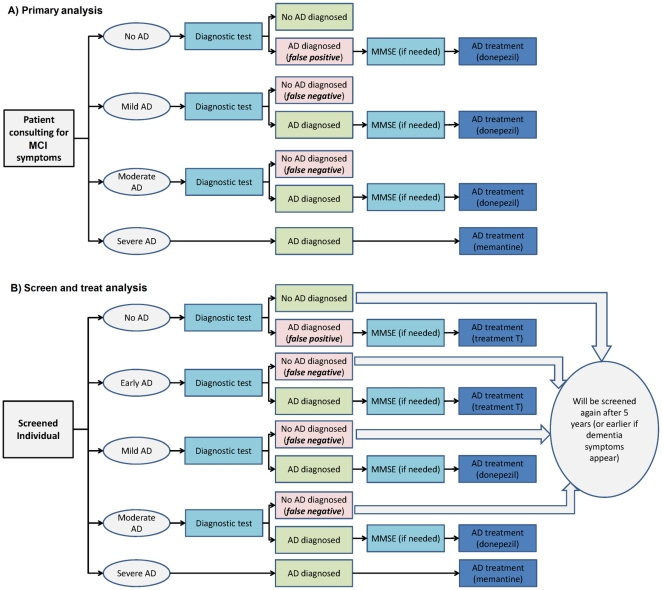
Decision tree for an individual tested for Alzheimer's disease (AD). Possible outcomes of the testing procedure are depicted as a function of the individual's health status for: (a) The primary scenario (testing of over-70 patients consulting for dementia symptoms). (b) The “screen and treat” scenario (systematic screening of the over-60 population). Depending on the investigated strategy, the generic “diagnostic test” mentioned in the trees may be standard diagnosis, standard MRI or MRI+CLP. When AD is diagnosed, the imaging procedures are followed by a cognition test (MMSE) in order to determine the disease stage. No test is performed in severe AD patients, who are assumed to be diagnosed directly.

Figure 1A depicts the tree showing the possible outcomes of using one of the investigated diagnostic tests in this scenario. The choice of a 3-year horizon for this analysis was motivated by data on the duration of efficacy of currently available AD treatments and mortality rates of over-70 dementia patients.

As part of the sensitivity analysis, we assumed that a new treatment had been developed which delayed significantly the course of AD at an early stage and that this treatment was offered to MCI patients diagnosed with AD but with high MMSE scores (who were either false positives or early AD patients).

#### 1.2. “Screen and treat” analyses

In the “screen and treat” analyses, it was assumed that a new treatment had been developed which delayed significantly the course of AD at an early stage, and that a national screening campaign targeted at individuals over 60 years-old took place. Screening could either be population-wide or limited to individuals carrying the ε4 allele of the apolipoprotein E gene (ApoE4), in whom the risk of AD has been shown to be significantly higher [16].

In this context, three diagnostic strategies were compared over a fifteen year period for a cohort of 60 year-old individuals taking part in the screening campaign:

the standard diagnosis strategy,
the standard MRI,
and the MRI+CLP strategy

Figure 1B depicts the tree showing the possible outcomes of the screening procedure with one of the investigated diagnostic tests in this scenario.

Following the initial screening, individuals who were not diagnosed as AD patients were screened again every 5 years, unless they developed dementia symptoms between these scheduled screenings, in which case they were immediately tested.

The choice of a 15 year horizon for this second analysis was motivated by the need to follow 60 year-old mostly healthy individuals through several screening campaigns.

### 2. Models of disease progression

We used a Markov model of the evolution of AD based on previous work [17] in which the disease evolved in 5 stages: no AD, mild AD, moderate AD, severe AD and death. For the screen and treat analyses, a 6^th^ stage was added for asymptomatic patients with early AD. We also distinguished patients that were taken care of at home from institutionalized patients, at each stage of AD. Initially, all patients in the cohort began in the community setting and had a probability in each cycle, conditional on disease state (and, for the 2d analysis, on age), of making a transition to nursing home care. File S1 and Figure S1 present the models we used in more detail.

Considering the natural history of AD, we chose to model disease progression using 6 month cycles. Transition probabilities over 6 months were obtained from the square root of the matrix of annual transition probabilities which were based on the literature [17], [18], [19] and available data on age-specific mortality rates [20] and age-specific AD incidence [4] in France.

Table S1 lists all model parameters, with their base-case values.

### 3. Available treatments

The model assumes that all patients who receive a diagnosis of probable Alzheimer disease receive treatment with donepezil, memantine or a hypothetical higher-efficacy drug.

If the patient is diagnosed at a mild or moderate stage, donepezil is prescribed, to be replaced by memantine when the patient has evolved to a severe stage of AD. If the patient is diagnosed when the disease is already severe, memantine is prescribed.

In addition, a hypothetical treatment T which is efficient at early stages of AD may be prescribed to patients who are diagnosed with AD but have high MMSE scores in the primary analysis, or to patients who are diagnosed with early AD in the “screen and treat” analyses.

Donepezil treatment has been shown to improve significantly memory and other cognitive functions in patients with mild to moderate AD [21], and to reduce the annual decline in cognition in these patients when compared with patients in a placebo group [22]; similarly, memantine treatment has been shown to cause a clinically noticeable reduction in deterioration over 28 weeks, compared with placebo, in patients with moderate-to-severe AD [23], [24]. It is to be noted that actually, donepezil or memantine help treat the symptoms of AD although there is no evidence that they modify the underlying pathology of the disease. On the basis of these data, we assumed that treatment modified transition probabilities between disease stages, both reducing the speed of AD progression and increasing the chance of symptom lessening (Table S1). For treatment T, we only assumed a decrease in the progression from early to mild and moderate stage AD, as we supposed that reversal to asymptomatic AD of mild stage patients was not possible. In the base-case analysis, these effects were assumed to be constant throughout the duration of treatment; long-term clinical studies suggest that treatment efficacy may last for up to 3 years [21], [25].

### 4. AD prevalence and incidence

In the primary analysis, the simulated cohort comprised 70 year old individuals who consulted for cognitive impairment symptoms. There is evidence that older persons with Mild Cognitive Impairment (MCI) feature neurobiological AD in 50% to 70% of the cases [26]. Based on this evidence and on data from a study at Massachusetts General Hospital [10], we estimated that 56% of the cohort patients had AD initially. The distribution between disease stages at this first consultation was estimated, based on a French study [27], at 55.9% mild stage AD, 39.9% moderate stage AD and 4.2% severe stage AD.

MCI is often a precursor to Alzheimer's dementia and the annual rate of development of AD for patients with MCI is 10 to 15% [26], [28]. Here, we assumed a 10%/year AD incidence in untreated patients of the cohort.

In the first “screen and treat” analysis, the cohort was representative from the French population of individuals over 60 years old, as it was assumed that a national screening was underway. Therefore the initial prevalence of AD (including early asymptomatic stage AD) in the cohort was assumed to be 1% [3]. We further assumed that there was an 8/7 ratio between the prevalences of early stage and mild stage AD [Personal communication, Pr. Verny, AP-HP, Paris, 2009]. The distribution between mild, moderate and severe stages was the same as in the primary analysis.

Age-specific AD incidence rates for individuals between 60 and 75 years old were chosen to be consistent with data from recent cohort studies [29], [30].

In the second “screen and treat” analysis, screening was restricted to individuals carrying the ε4 allele of the apolipoprotein E gene (ApoE4). Based on earlier studies showing significantly higher risk of AD occurrence in ApoE4 individuals [16], we assumed that age-specific incidence rates were doubled.

### 5. Diagnostic tests

The standard diagnostic strategy of AD was assumed to comprise a detailed history of the patient, an assessment of cognition and functional status using a questionnaire test such as the MMSE, and laboratory testing. Other strategies combined MRI (with or without a new contrast agent) for AD diagnosis with a questionnaire test such as the MMSE aimed at determining the stage of the disease.

In the case of severe AD, it was assumed that dementia symptoms allowed direct diagnosis over a consultation (without need for a diagnostic test). Hence, all severe stage AD patients were assumed to be diagnosed and to receive treatment.

As in [10], we estimated the sensitivity of the standard diagnostic tests at 75% and their specificity at 90%; in early stage AD, we assumed that patients were asymptomatic and that the standard diagnostic therefore performed as it did in non-AD patients, declaring only 10% ( = 1−0.9) of them as having AD. Based on data from a clinical study, we also hypothesized a sensitivity of 50% in early stage, 88% in mild stage and 95% in moderate stage AD, as well as a 96% specificity, for standard MRI diagnosis [31].

Regarding the hypothetical diagnostic test using MRI with the new contrast agent (MRI+CLP), we based our assumptions on available data on PET-scan amyloid imaging. As studies on amyloid plaques suggest that amyloid deposition reaches a plateau by the early clinical stages of Alzheimer's disease (amyloid cascade hypothesis), we assumed the sensitivity of the MRI+CLP diagnostic test to be independent of the disease stage. In one study on PET tracers, the sensitivity and specificity were estimated at 90% [32]; in another, a 95% sensitivity and 83% specificity were found [33]. Here, we assumed a 96% sensitivity and 87% specificity for MRI+CLP.

### 6. Costs and effectiveness

All costs were measured at their 2009 level.

#### 6.1. Costs of diagnostic tests

The cost of the standard diagnosis was computed as the sum of the cost of a specialist consultation in France, estimated at 55€ [34], that of mental state evaluation tests, estimated at 69€ [35], and that of standard laboratory tests, estimated at 50€ [36]. The cost of MRI was obtained from the “Classification Commune des Actes Médicaux”, a fixed-costs scale of medical procedures based on practitioners' fees, fixed costs for the medical procedures themselves, and fixed costs for operating the equipment [35]. Finally, we estimated the cost of the new contrast product for MRI at 250€ [Personal Communication, Guerbet company, Paris, 2009].

#### 6.2. Costs of AD follow-up

Diagnosed AD patients were assumed to have a follow-up consultation every 6 month with an AD specialist. These specialist consultations were estimated at 41€ [34].

#### 6.3. Costs of treatment

We used prices for generic AD drugs, that is, 572€ per 6-month period for generic donepezil (Aricept) and 286€ per 6-month period for generic memantine (Ebixa) [37]. We further assumed that the hypothetical new drug that would be efficient in early stage AD would have a cost similar to that of generic donepezil.

#### 6.4. Costs of care

We took into account both living and care costs. For institutionalized patients, living costs included hostel costs, based on a French study of AD patients [18], and costs of care included caregiver wages. In an earlier study, caregivers were estimated to spend 517 hours over a 3 month period caring for moderate-to-severe AD patients [38]. Here, we therefore computed costs associated with caregivers over 6 months by multiplying hourly wages (estimated at 13 €/h for professional caregivers) with 1034 hours.

For patients living at home, “basic” costs included living and medical expenditures [39], based on a French study of home-cared AD patients and controls; the cost of care by unprofessional caregivers was also estimated as an opportunity cost. In order to do this, we valued informal care at a conservative value of 8.4 €/h, and we assumed that these unpaid caregivers spent the same amount of time caring for AD patients than professional caregivers, that is, 1034 hours over a 6 month cycle.

#### 6.5. Indirect costs

In this study, we took into account several indirect costs of AD. First, we assigned an opportunity cost to unprofessional caregivers who took care of AD patients leaving at home, as described before.

Second, we took into account the burden of AD care on the health state of unprofessional caregivers. Based on an earlier study [38], 35% of AD caregivers take medication related to their activity; they have high rates of depression and anxiety, as well as high overall morbidity and mortality rates, compared to non-AD caregiver controls [40]. We estimated the cost of this health impact as that as of a weekly psychiatrist consultation, plus that of an antidepressant treatment, for 35% of unprofessional caregivers.

Finally, we also evaluated the cost associated with the loss of productivity of AD patients. This is generally not done in cost-effectiveness studies of AD, since AD patients are for the most part retired. However, recent data shows that pensioners are becoming more and more involved in volunteer activities within nonprofit organizations (NPOs), as well as performing informal volunteer activities [41].

Here, based on French data [42], we estimated that 60 to 75 year-old individuals performed on average 63.8 hours of informal volunteering activities over a 6 month period – mostly within the family sphere, such as childcare for instance. This was valued at 7.7€/hour, which was the minimum wage in France in 2009, and multiplied by an “efficiency coefficient” of 0.7 to arbitrarily take into account the reduced productivity in older individuals.

Similarly, we estimated that 50% of 60 to 75 year-old individuals are involved in NPOs, with a mean of 12 hours of volunteer activities per month. On average, we hence estimated that 60 to 75 year-old individuals performed a total of 36 hours of volunteer activities within NPOs over a 6 month period [43], which were valued at 7.9€/hour and multiplied by the aforementioned 0.7 efficiency coefficient.

We added the resulting estimated productivity benefit of a French pensioner to our analyses as a cost associated to AD, in full for moderate-to-severe patients and multiplied by 0.6 for mild AD patients (assuming that mild AD only reduces productivity by 40%).

#### 6.6. Effectiveness

We estimated quality-of-life weights (QALYs) for over-60 patients without Alzheimer disease at 0.826 on a scale of 0 to 1, on the basis of the mean of time trade-off scores for men and women aged 65–84 years old published in a study of health outcomes in the general population [44].

Quality-of-life weights for patients with Alzheimer disease at each disease stage and care setting (institution or community) were based on previously published Health Utilities Index Mark 2 (HUI:2) scores [17].

### 7. Sensitivity analysis

The ranges investigated in the Sensitivity Analysis are summarized in Table S1, along with the data sources. We performed both univariate and multivariate sensitivity analyses. For the multivariate analyses, we performed a Monte-Carlo simulation with 10.000 trials, using *a priori* triangular distributions for model parameters. We then identified the most influential parameters in the cost-effectiveness of the MRI+CLP strategy by calculating the partial rank correlation coefficient (PRCC) between each input parameter and the ICER of this strategy and assessing their statistical significance.

#### 7.1. Drug effects and prices

We investigated the impact of the introduction of new drugs, which would have the same costs and indications as donepezil and memantine, respectively, but with varying efficacy: the probabilities of transitions under treatment from mild to moderate or moderate to severe stage were further reduced by a factor (f_mM_ or f_MS_) ranging from 0.5 to 1, and the probabilities of transitions under treatment from moderate to mild or severe to moderate stage were further increased by a factor (f_Mm_ or f_SM_) ranging from 1 to 2 [17]. For simplicity reasons, we also summarized these four avenues for improvement of current AD treatment through a single parameter f, assuming a linear relationship between the multiplying factors: 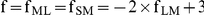


Regarding the hypothetical drug efficient for early stage AD (treatment T), we investigated both more and less efficient drugs, with probabilities of progression from early to mild and moderate stage AD under treatment T ranging from 0 to their values without treatment T; f_T_ was the reduction factor applied to these transition probabilities due to treatment T (in [0–1]).

The assumed cost for a 6-month cure with treatment T was also varied, between 0 and 1000€.

#### 7.2. Diagnostic tests characteristics

We investigated sensitivities from 0.9 to 1 and specificities from 0.7 to 1 in early to moderate stage AD patients for the hypothetical diagnostic test using MRI with the CLP contrast product. We also investigated sensitivities ranging from 0.75 to 0.90 for the standard diagnosis in moderate stage AD.

In the “screen and treat” analyses, we investigated sensitivities of standard MRI between 0.1 and 0.5 for early stage AD.

#### 7.3. Disease progression

To model faster or slower progression of AD, we investigated state transition probabilities in our Markov model ranging from 10% lower to 10% higher than their base-case values.

#### 7.4. Initial distribution of patients and prevalence

In the primary analysis, we investigated AD prevalences among consulting individuals ranging from 50 to 70%. For each fixed prevalence, we varied the proportion of patients with mild AD between 50 and 75% of all AD patients; the ratio between moderate and severe AD prevalence remained the same, that is, 10 moderate stage patients for 1 severe stage patient.

In the “screen and treat” analyses, we investigated initial AD prevalences among screened individuals ranging from 1 to 10%. For each fixed prevalence, we varied the proportion of patients with early asymptomatic AD from 30 to 75% of all AD patients; the distribution of mild to severe stages remained the same.

#### 7.5. Discount rate

As the “screen and treat” analyses spanned a 15 year period, we assessed the impact of variations in the assumed discount rate for costs and QALYs, from 0 to 10% per year.

#### 7.6. Frequency of screenings

In the “screen and treat” analyses, we investigated the impact of varying durations between screening campaigns, ranging from one to ten years.

## Results

### 1. Primary analysis

#### 1.1. Base Case

The first part of Table 2 summarizes the results of the primary cost-effectiveness analysis in the base case; in the second part of Table 2, hypothetical treatment T is offered to all MCI patients diagnosed with AD but with high MMSE scores. In both cases, the standard diagnosis strategy was dominated by the standard MRI strategy (more costly and less effective).

**Table 2:** Results of the primary analysis (base-case hypothesis): computed cost, efficacy and cost-effectiveness (C/E) ratio of the standard diagnosis, standard MRI and MRI+CLP strategies, and incremental cost-effectiveness ratios (ICER) of the MRI+CLP strategy as compared with the standard MRI strategy.

Strategy	Cost (in €)	Efficacy (in QALYs)	C/E	ICER
**In the current context**				
Standard diagnosis	36 294	1.7663	20 548	Dominated
Standard MRI	36 131	1.7710	20 401	
MRI+CLP	36 313	1.7731	20 480	88 439 €/QALY
**Assuming hypothetical treatment T has been made available**				
Standard diagnosis	36 260	1.7668	20 523	Dominated
Standard MRI	36 117	1.7712	20 391	
MRI+CLP	36 268	1.7737	20 447	60 923 €/QALY

Cost and effectiveness increased from standard MRI to MRI+CLP strategies. In the base-case, the ICER of the MRI+CLP strategy was higher than the French willingness-to-pay threshold (estimated at 76 171 €/QALY). Hence, standard MRI was found to be the preferred strategy. However, assuming that treatment T had been made available led to a lower ICER for MRI+CLP, making it the preferred strategy.

#### 1.2. Sensitivity analysis

Detailed results of the univariate sensitivity analysis are presented in Table S2 (without treatment T). Assuming either more effective AD treatments, higher speed of AD progression, a larger initial AD prevalence in 70 year-old MCI individuals, a larger initial portion of mild AD, higher sensitivity or specificity of MRI+CLP diagnosis or a lower cost of the CLP product could lead to MRI+CLP becoming the preferred strategy, with an ICER lower than the willingness-to-pay threshold.

Table 3 (columns 1 and 2) provides partial rank correlation coefficients (PRCC) between input values and the ICER of the MRI+CLP strategy (compared with the standard MRI strategy, without treatment T). Only parameters whose PRCC with this ICER is statistically significant at the confidence level of 5% or lower are shown, namely: the speed of disease progression; the initial AD prevalence in the studied population; the initial portion of mild stage AD; the discount rate; the sensitivity and specificity of the MRI+CLP diagnostic test; and the cost of the CLP contrast agent.

**Table 3:** Partial rank correlation coefficients (PRCC) between input values and the ICER of the MRI+CLP strategy (compared with the preferred strategy).

Primary cost-effectiveness analysis		“Screen and treat” cost-effectiveness analysis (population-wide screening)	
Parameter	PRCC	Parameter	PRCC
Speed of disease progression	−0,41	Initial prevalence of AD	−0,86
Initial prevalence of AD	−0,51	Initial portion of early AD	−0,15
Initial portion of mild AD	−0,26	Sensitivity of MRI+CLP test	−0,17
Sensitivity of MRI+CLP test	−0,92	Specificity of MRI+CLP test	−0,64
Specificity of MRI+CLP test	−0,73	Discount rate	0,29
Discount rate	0,19	Cost of the CLP contrast agent	0,41
Cost of the CLP contrast agent	0,79	Cost of treatment T	0,78
		Impact of treatment T (from high to low)	0,41
		Speed of disease progression	−0,23
		Sensitivity of standard MRI in early AD	0,51

All PRCCs are statistically significant at the confidence level of 5%. A higher absolute value of PRCC indicates a strong relationship between that parameter and the ICER; a positive (resp. negative) value of PRCC implies that the value of the ICER increases (resp. decreases) when the value of the input increases.

**Figure 2:**
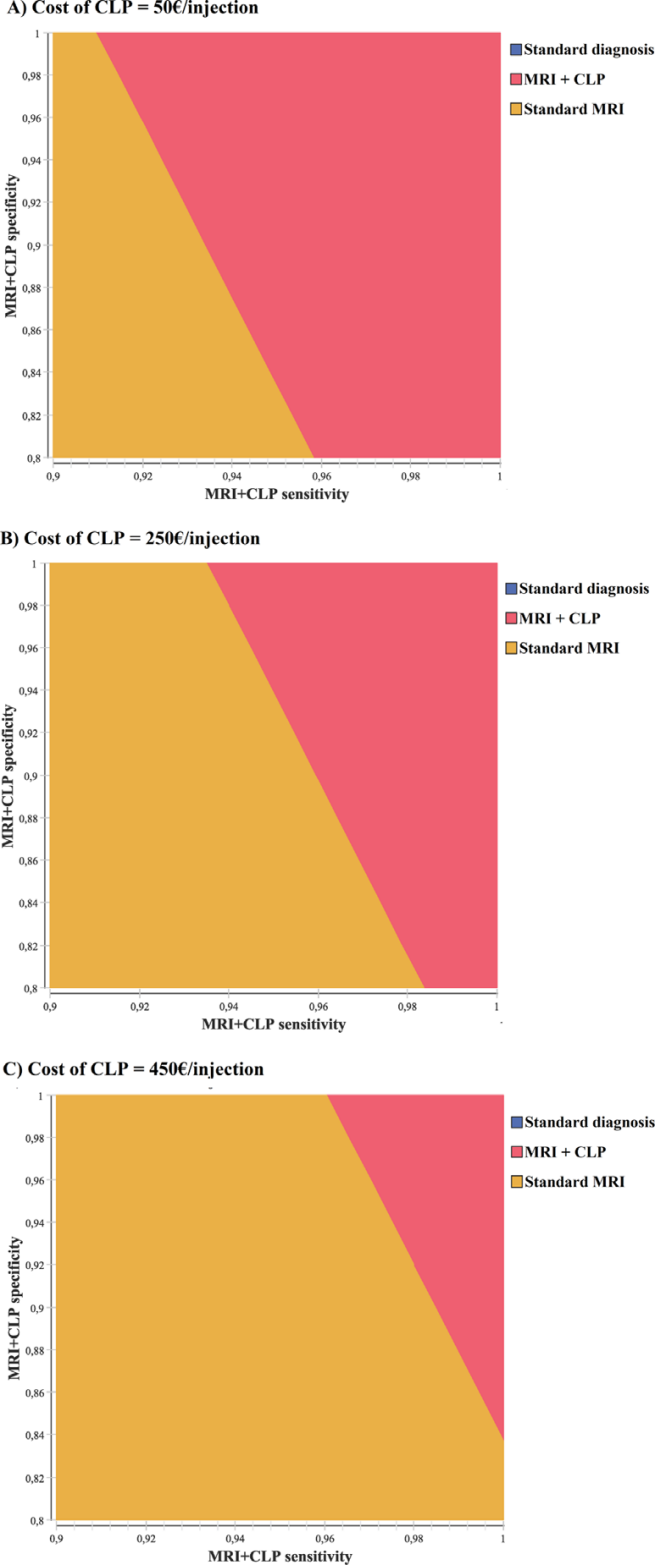
Results of the primary analysis: multivariate sensitivity analysis. The strategy with maximum net monetary benefit is depicted as a function of the assumed sensitivity and specificity of the MRI+CLP diagnostic test, for assumed costs of the CLP contrast agent between 0 and 500€ per injection (in the absence of treatment T): (a) Cost of the CLP contrast agent at 50 €/injection. (b) Cost of the CLP contrast agent at 250 €/injection. (c) Cost of the CLP contrast agent over 450 €/injection.

In Figure 2, the sensitivity of our conclusions on the MRI+CLP diagnostic test characteristics (sensitivity, specificity and cost) is assessed. MRI+CLP is the preferred strategy in a wide array of scenarios, including some assuming lower sensitivity or specificity or higher cost than in the base-case.

Figure S2 provides the distribution of incremental cost-effectiveness ratios for the MRI+CLP strategy, compared with standard diagnosis, as well as an acceptability curve depicting the probability for each of the three investigated strategies to be preferred as a function of the willingness-to-pay threshold. For willingness-to-pay thresholds larger than 90 000€/QALY, MRI+CLP becomes the preferred strategy.

With the assumed WTP threshold at 76 171€/QALY, the probability that MRI+CLP was the preferred strategy increased from 43% to 64% when treatment T was assumed to be available (data not shown). Figure S3 investigates the impact of the efficacy and cost of treatment T on these results.

### 2. “Screen and treat” analyses

#### 2.1. Base Case

Table 4 summarizes the results of the “screen and treat” cost-effectiveness analyses in the base case; in the first part, the whole over-60 population is screened, while in the second part, the screening is targeted at ApoE4 individuals. In both cases, the standard diagnosis strategy was dominated by the standard MRI strategy.

**Table 4:** Results of the “screen and treat” analyses (base-case hypothesis): computed cost, efficacy and cost-effectiveness (C/E) ratio of the standard diagnosis, standard MRI and MRI+CLP strategies, and incremental cost-effectiveness ratio (ICER) of the MRI+CLP strategy as compared with the standard MRI strategy.

Strategy	Cost (in €)	Efficacy (in QALYs)	C/E	ICER
**Population-wide screening**				
Standard diagnosis	43 559	8.0722	5 396	Dominated
Standard MRI	43 009	8.0732	5 327	
MRI+CLP	44 945	8.0752	5 566	991 972 €/QALY
**Screening targeted at ApoE4 individuals**				
Standard diagnosis	44 711	8.0377	5 563	Dominated
Standard MRI	44 180	8.0386	5 496	
MRI+CLP	46 075	8.0415	5 730	641 326 €/QALY

Although overall computed costs and QALYs were lower when assuming a screening targeted at the ApoE4 population, the main base-case results were similar in both our screening analyses. The MRI+CLP strategy was both more costly and more efficient than the standard MRI strategy. Its ICER, compared with the standard MRI strategy, was much higher than the French willingness-to-pay estimated at 76 171 €/QALY. Therefore, standard MRI was the preferred strategy in the base case.

#### 2.2. Sensitivity analysis

Detailed results of the univariate sensitivity analysis are presented in Table S3 for the population-wide screening scenario. For an assumed specificity of MRI+CLP higher than 98%, MRI+CLP became the preferred strategy; it was strongly dominant for specificities over 99%. No other individual model parameter had enough impact on our results to make MRI+CLP the preferred strategy.

Table 3 (columns 3 and 4) provides partial rank correlation coefficients (PRCC) between inputs values and the ICER of the MRI+CLP strategy (compared with the standard MRI strategy), for the population-wide screening scenario. Only parameters whose PRCC with this ICER is statistically significant at the confidence level of 5% or lower are shown, namely: the initial AD prevalence in the studied population; the specificity of the MRI+CLP diagnostic test; and the efficacy and cost of new treatment T.

**Figure 3:**
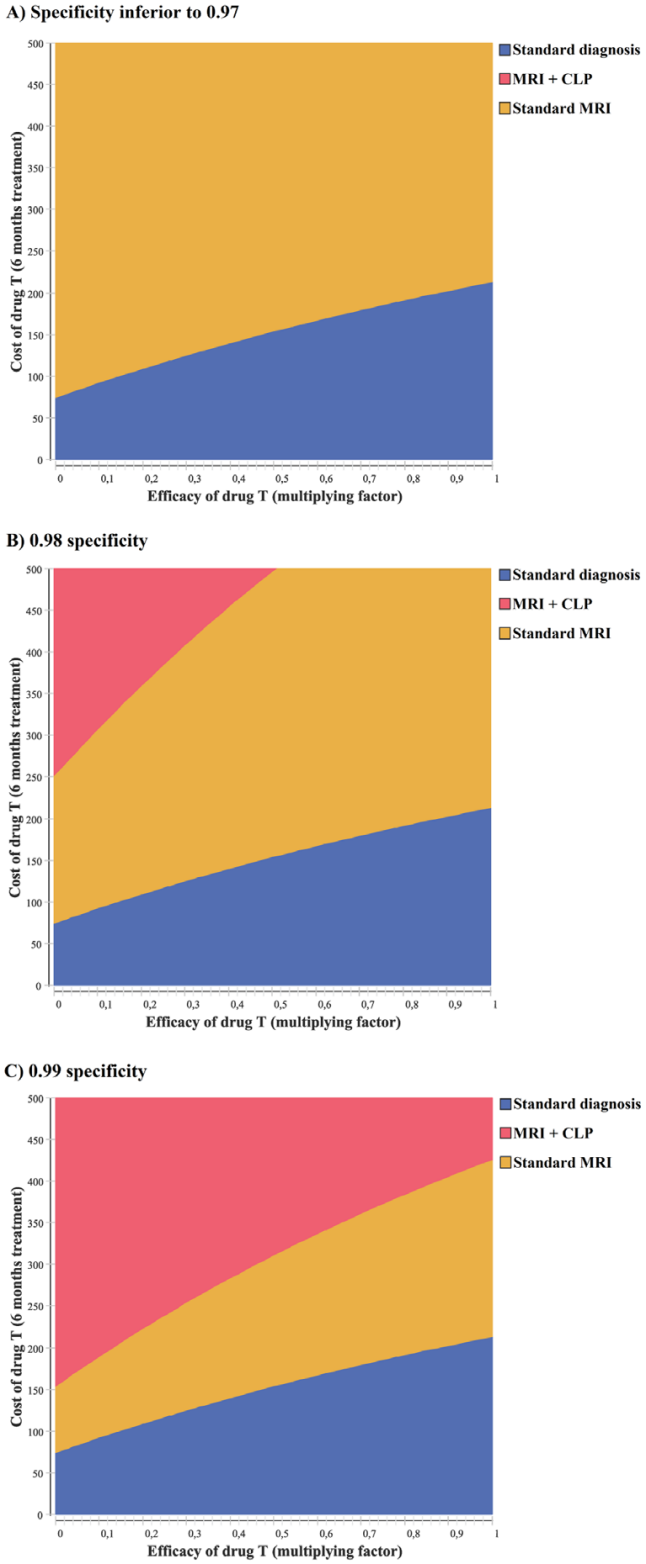
Results of the “screen and treat” (population-wide screening) analysis: multivariate sensitivity analysis. The strategy with maximum net monetary benefit is depicted as a function of the assumed efficacy and cost of the hypothetical new drug T, for assumed specificities of the MRI+CLP diagnosis test between 0.80 and 0.99. The efficacy of treatment T is expressed as a 0-to-1 ratio between assumed probabilities of transition from early stage AD with and without treatment T; 0 corresponds to maximum efficacy and 1 to no efficacy (in the base case, f_T_ = 0.5: 50% reduction). Only costs lower than the base-case cost of 500€ per 6-month treatment are investigated here. (a) specificity for MRI+CLP inferior to 0.97 (including base case). (b) 0.98 specificity for MRI+CLP. (c) 0.99 specificity for MRI+CLP.

**Figure 4:**
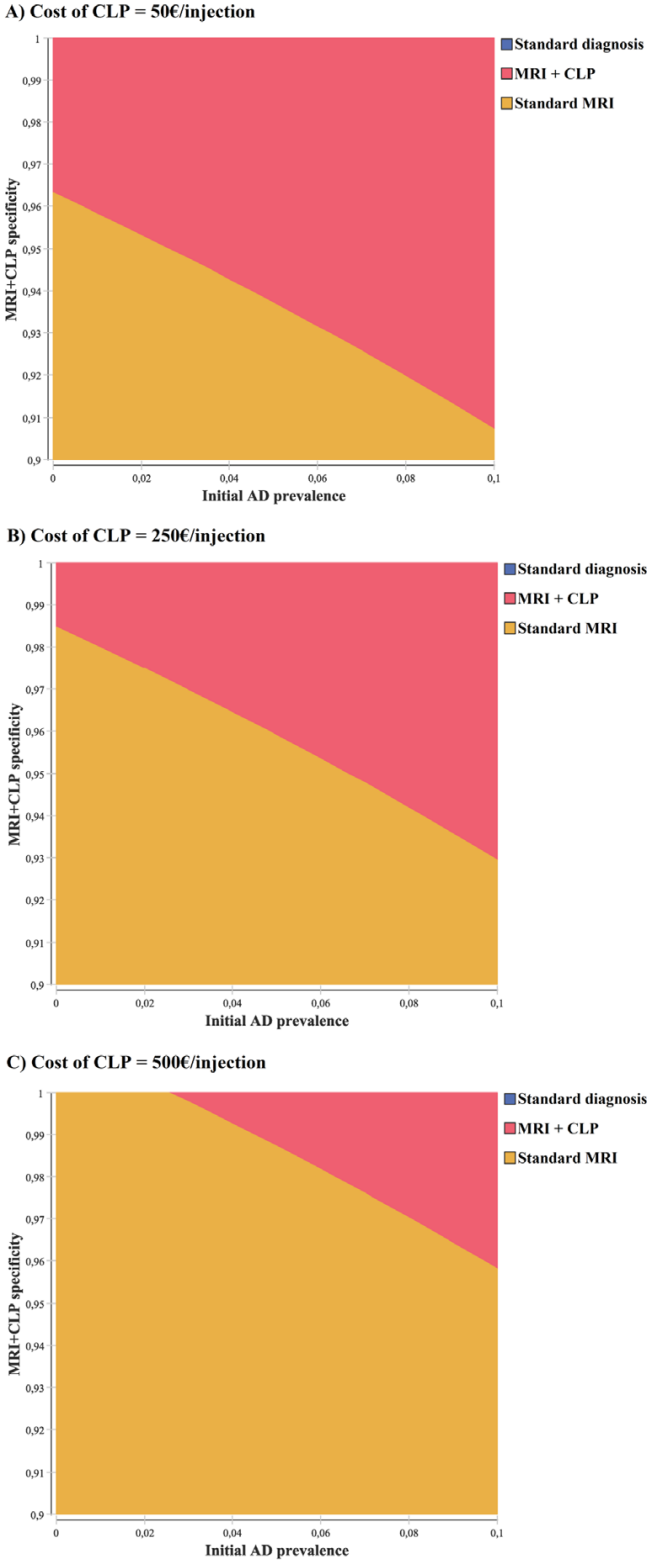
Results of the “screen and treat” (population-wide screening) analysis: multivariate sensitivity analysis. The strategy with maximum net monetary benefit is depicted as a function of the assumed prevalence of AD in the general over-60 population (between 0 and 10%) and specificity of the MRI+CLP diagnostic test between 0.90 and 1, for assumed costs of the CLP contrast agent between 0 and 500€ per injection: (a) Cost of the CLP contrast agent at 50 €/injection. (b) Cost of the CLP contrast agent at 250 €/injection (base-case). (c) Cost of the CLP contrast agent at 500 €/injection.

Figure 3 depicts results from a multivariate analysis performed on the efficacy and cost of the hypothetical new treatment and MRI+CLP specificity, while Figure 4 depicts results from a multivariate analysis performed on the initial prevalence of AD in the general over-60 population, the specificity of MRI+CLP diagnosis and the assumed cost of the CLP contrast agent. Several combinations of these parameters allow for MRI+CLP strategy dominance, but all include high specificity of MRI+CLP diagnosis. For instance:

assuming 98% specificity for MRI+CLP diagnosis and that treatment T reduces AD progression from the early stage by half, MRI+CLP is the preferred strategy for the base-case cost of treatment T (500€/6 months of treatment)
assuming an initial AD prevalence of 4% and the base-case cost of 250€ per injection for the CLP contrast agent, MRI+CLP is the preferred strategy as long as MRI+CLP diagnosis is more than 97% specific.

Figure S4 provides the distribution of incremental cost-effectiveness ratios for the MRI+CLP strategy, compared with standard MRI, as well as an acceptability curve depicting the probability for the MRI+CLP strategy to be the preferred strategy as a function of the willingness-to-pay threshold. This probability remains lower than 4% even assuming a willingness-to-pay at 200 000€/QALY.

When assuming a screening targeted at the ApoE4 population, the probability that MRI+CLP was the preferred strategy increased slightly, reaching 5% for a 200 000 €/QALY willingness-to-pay threshold (data not shown).

## Discussion

Here, we investigated the cost-effectiveness of a new diagnostic tool for AD allowing early diagnosis in two different contexts. First, we assumed that this new diagnostic tool would be made available to the same individuals who are currently offered other diagnostic tests. Second, we hypothesized that a treatment with significant efficacy in early stage AD was developed, and that, as a consequence, a national screening campaign was put into place, using either the currently available diagnostic tests or the new diagnostic test.

We found that in both analyses, the preferred base-case strategy was the standard MRI strategy. However, multivariate sensitivity analyses showed that, while in the primary analysis combining MRI with a new contrast product could prove the preferred strategy under a wide array of realistic scenarios, the probability of this happening was inferior to 5% in the hypothesis of a national screening campaign. Even assuming the availability of a low-cost highly efficient treatment in early AD, novel contrast agents would need to have very high specificity to be cost-effective when used for systematic screening of the entire population.

### 1. Models of disease progression

We used Markov models of AD progression. Although widely used in similar cost-effectiveness analyses [10], [11], [17], [18], [45], [46], this approach presents several limitations. First, it has been shown that using time-independent transition probabilities may lead to either overestimate or underestimate disease progression, depending on the AD stage [47]. Second, in order for the model complexity to remain manageable, we were led to limit the number of states. In order to take into account indirect consequences of AD, such as the loss of productivity of AD patients in their volunteer activities, we incorporated them as aggregate factors in the computation of costs.

Using other modeling approaches, such as discrete events simulation, would allow a more detailed and realistic description of AD progression and, in turn, derive more reliable conclusions [12], [48].

### 2. Assumed properties of the MRI+CLP diagnostic test

Because the new CLP contrast agent for MRI is still under development, assumptions had to be made regarding its sensitivity and specificity for diagnosing AD. As mentioned in the Methods, we based these assumptions in part on available data on PET-scan used with amyloid markers [32], [33]. We also investigated relatively wide ranges for these characteristics in our sensitivity analyses.

Regarding the specificity, it should be noted that amyloid plaques have been detected in 10 to 30% of otherwise apparently normal elderly subjects. This may limit the capacity of MRI+CLP to correctly identify healthy subjects, which is why we investigated specificities as low as 0.70 in our sensitivity analyses. However, several studies also suggest that the presence of amyloid plaques is associated with declining cognitive test scores and with structural and functional brain changes suggestive of early AD [49]. In two recent follow-up studies, about one third of patients with mild cognitive impairment in whom amyloid plaques were detected converted to AD over the following 2 years [50], [51]. It could therefore be argued that at least part of these 10–30% amyloid-positive individuals may not be “false positive”, but very early stage AD patients.

Figure S5 illustrates the influence of false positives and false negatives associated with the MRI+CLP diagnosis in terms of additional cost per QALY in both our primary analysis and our “screen and treat” analysis.

### 3. Included costs

In this study, we chose to include indirect costs in addition to direct costs. The importance of these indirect costs, which include costs associated with the loss of productivity of AD patients and costs associated with informal caregiving at the homes of AD patients, has indeed been underlined in several recent studies, which showed that they may dominate direct costs of care in early stages of the disease [52]. As a consequence, it has been suggested in recent reviews that future cost-effectiveness studies should take into account such indirect costs [48], [53].

### 4. Results and limits of the primary analysis

The results we obtained in our primary analysis are similar to those from previously published cost-efficacy studies, in the sense that in the base-case, adding a new marker to MRI was not cost-effective [10], [11]. However, in our multivariate sensitivity analysis, there was a high probability of MRI+CLP becoming the preferred strategy.

In addition, it is to be noted that we found the standard MRI strategy to dominate the standard diagnosis strategy, whereas earlier studies found very high ICERs for similar imaging strategies [10], [11]. This may be due to the fact that in these earlier studies, MRI was supposed to come as an addition to the standard diagnosis procedure, which included a detailed history, a questionnaire test such as the MMSE, laboratory testing and nonenhanced CT imaging. In our study, the standard MRI strategy only comprised the initial specialist consultation, the MMSE and MRI itself.

Because of the high level of uncertainty surrounding the assumed sensitivity and specificity of both standard diagnosis and standard MRI, we also explored how our results changed when assuming higher sensitivity and specificity for the standard diagnosis, or lower sensitivity and specificity for standard MRI. Irrespective of the assumed specificity, the standard diagnosis strategy remained dominated by the standard MRI strategy. However, as shown in Figure S6, standard diagnosis could become preferable to standard MRI under a wide array of combinations of lower sensitivity for standard MRI and higher sensitivity for standard diagnosis in moderate AD.

All in all, it should be underlined here that comparing standard MRI to the current standard diagnosis strategy for AD was not the focus of this study and that our results should not be taken as evidence that large-scale screening using standard MRI should be undertaken.

Several limitations of this analysis lie with the assumptions we made on AD treatment.

First, we neglected all adverse effects from the treatments. Including a cost associated with such adverse effects in treated individuals would lead to reduced ICERs for all strategies. As costs would be expected to increase while QALYs remained unchanged, the ICER of the MRI+CLP strategy should only increase.

Second, we assumed that all diagnosed individuals were treated. This is in reality probably false, as AD patients may end up rejecting treatment or not taking it properly.

Finally, another limitation of this analysis is that we did not take into account the psychological impact of early diagnosis of AD in the absence of efficient treatment at this stage. Including this factor would increase the cost associated to the MRI+CLP strategy, and in turn its ICER.

### 5. Results and limits of the “screen and treat” analyses

In our “screen and treat” analyses, we investigated a hypothetical future in which new treatments effective in early stage AD were available. To our knowledge, ours is the first study to do so, although earlier works investigated improvements in currently available treatments for mild to severe stage AD [10], [11]. Innovatively, this second analysis also included a community-wide screening program and repeated rounds of testing. Indeed, if such treatments were to become available, population screening programs – which are not currently recommended by any health Agency – would have to be discussed.

Because the investigated context was hypothetical, we had to make several assumptions which influenced our results. First, we had to estimate the prevalence of AD in the general over-60 population, including asymptomatic cases. There is obviously no data to document this prevalence, and we chose a rather conservative value of 1%; the actual figure may be well over this value and we investigated prevalences up to 10% in our sensitivity analysis. Our base-case analysis was a worst-case scenario for the MRI+CLP strategy, which included the only diagnosis tool adapted to early AD.

Second, as both the new treatment and the new diagnosis test were hypothetical, we chose values for their base-case characteristics based on personal communications with AD specialists and teams currently working on the development of CLP tracers [Personal Communication, Guerbet company, Paris, 2009], and investigated wide ranges of variation for these values in our sensitivity analysis. It is to be noted that the new treatment T may well be more expensive than 1000€, the top limit of our investigated range; however, as shown by our results, MRI+CLP would then systematically not prove cost-effective, irrespective of other characteristics.

Third, the participation rate to the screening campaign was unknown, and assumed to be 100%. Based on observed data from similar screening campaigns, actual rates are more around 60% at best, meaning that the screened population may not be representative of the general over-60 population. Also, being screened positive for AD may have a psychological impact, especially in asymptomatic patients. We chose not to take into account this impact in our analysis, considering that it should be reduced by the availability of a treatment efficient from early AD. Future studies should model screening participation and impact more realistically and investigate the potential repercussions.

Finally, this second analysis suffered from the same limitations related to AD treatment as the first. Including adverse effects of treatments in this analysis would increase the cost of the MRI+CLP strategy. Moreover, as the time horizon for this analysis was 15 years, it would have been interesting to investigate the impact of varying durations of treatment efficacy. This will be made easier in future years when more data on the long-term impact of AD treatment becomes available.

Because there is evidence that individuals carrying the ApoE4 gene are at increased risk for AD, we felt it was pertinent to investigate a scenario under which screening was targeted at these individuals. It should be noted however, that, in order to fully investigate such a scenario, other factors may have to be taken into account. Indeed, some epidemiological studies suggest that the ApoE4 genotype increases mortality rates, in particular cardiovascular mortality in older individuals [54], hence age-specific mortality rates would need to be obtained for ApoE4 individuals. In addition, amyloid plaque density in cognitively healthy adults has been shown to be higher in ApoE4 carriers [55]. Depending on the interpretation of this finding, this may have different implications for a diagnostic tool based on the detection of amyloid plaques. On the one hand, it could be assumed that ApoE4 carriage increases the speed of amyloid plaque accumulation in AD patients; in that case, MRI+CLP would have better sensitivity in early stage AD in ApoE4 carriers. On the other hand, it could also be assumed that the ApoE4 genotype increases the density of amyloid plaques irrespective of AD; in that case, MRI+CLP would be expected to have lower specificity in ApoE4 carriers.

### 6. Conclusions

Assuming that a treatment with proven efficacy in early AD becomes available, as well as a diagnostic test allowing early detection of the disease, the issue of screening the population will arise. Our study suggests that, in order for this screening to be cost-effective, key parameters are the specificity of the new diagnostic test and the cost and effectiveness of the new treatment. These preliminary results ought to be taken into account in the currently underway research on early detection and treatment of AD, including work on β-amyloid plaques detection and elimination.

When this research yields results, a new cost-effectiveness analysis should be performed in order to evaluate the available tools with observed data.

## Supporting Information

Figure S1 **Markov models of Alzheimer's disease progression.** All states are further subdivided in two, for individuals living at home vs. inside an institution (retirement or nursing home). A) Model for the primary analysis. B) Model for the “screen and treat” analyses.
(TIF)

Figure S2 **Multivariate sensitivity analysis of the primary cost-effectiveness study.** A) Distribution of incremental cost-effectiveness ratios (ICER) of the MRI+CLP strategy, compared to the standard diagnosis strategy. B) Acceptability curve: probability that each strategy either is dominant or has an ICER inferior to the willingness-to-pay, as a function of the willingness-to-pay threshold.
(TIF)

Figure S3 **Multivariate sensitivity analysis of the primary cost-effectiveness study with treatment T.** The strategy with maximum net monetary benefit is depicted as a function of the assumed efficacy and cost of the hypothetical new drug T. The efficacy of treatment T is expressed as a 0-to-1 ratio between assumed probabilities of transition from early stage AD with and without treatment T; 0 corresponds to maximum efficacy and 1 to no efficacy (in the base case, f_T_ = 0.5: 50% reduction).
(TIF)

Figure S4 **Multivariate sensitivity analysis of the “screen and treat” (population-wide screening) cost-effectiveness study.** A) Distribution of incremental cost-effectiveness ratios (ICER) of the MRI+CLP strategy, compared to the standard MRI strategy. B) Acceptability curve: probability that the MRI+CLP strategy either is dominant or has an ICER inferior to the willingness-to-pay, as a function of the willingness-to-pay threshold.
(TIF)

Figure S5 **Analysis of the influence of MRI+CLP false positives and false negatives on the cost-effectiveness of the MRI+CLP strategies (tornado diagram).** The range of incremental cost-effectiveness ratios (ICER) of the MRI+CLP strategy (compared to the standard MRI strategy) in A) the primary analysis and B) the “screen and treat” analysis is depicted for MRI+CLP sensitivities ranging from 0.9 to 1 and specificities ranging from 0.7 to 1. A sensitivity lower than 0.96 (base-case value) implies a higher risk of false negatives (FN, dotted green bars) than in the base-case. A specificity lower than 0.87 (base-case value) implies a higher risk of false positives (FP, dotted yellow bars) than in the base-case.
(TIF)

Figure S6 **Multivariate sensitivity analysis of the results of the primary cost-effectiveness study on standard MRI vs. standard diagnosis.** The strategy with maximum net monetary benefit among these two strategies is depicted as a function of the assumed sensitivity of the standard MRI diagnostic test in mild and moderate AD, for an assumed sensitivity of the standard diagnosis strategy in moderate AD between 0.75 and 0.85 (in the absence of CLP and treatment T). A) 0.75 sensitivity in moderate AD for standard diagnosis (base-case value). B) 0.85 sensitivity in moderate AD for standard diagnosis.
(TIF)

File S1 **Models of disease progression.**
(DOCX)

Table S1 **Model parameters: base-case values and ranges investigated in the sensitivity analyses.**
(DOCX)

Table S2 **Univariate sensitivity analysis: incremental cost-effectiveness ratio (ICER) of the MRI+CLP strategy, compared to the reference strategy, in the primary cost-effectiveness analysis, depending on the values of model parameters.**
(DOCX)

Table S3 **Univariate sensitivity analysis: ICER of the MRI+CLP strategy compared with the reference strategy, in the universal “screen and treat” cost-effectiveness analysis, depending on the values of model parameters.**
(DOCX)
