# Supplementary figures and images for: Cost-Effectiveness of Magnetic Resonance Imaging with a New Contrast Agent for the Early Diagnosis of Alzheimer's Disease
Source: PLoS One. 2012 Apr 20;7(4):e35559. doi: 10.1371/journal.pone.0035559 (PMC3332046; doi:10.1371/journal.pone.0035559)

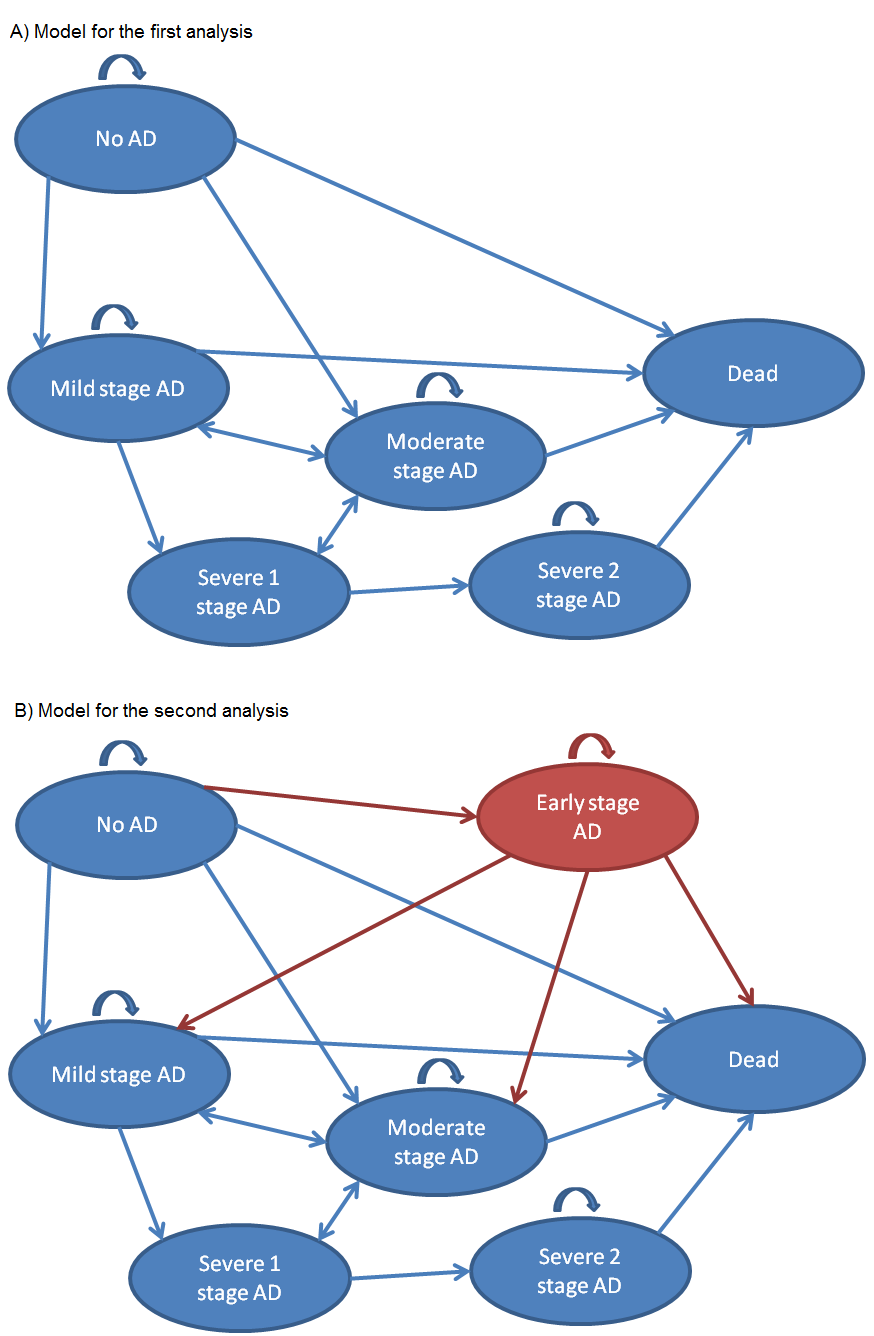

Supplement: Figure S1 — Markov models of Alzheimer's disease progression. All states are further subdivided in two, for individuals living at home vs. inside an institution (retirement or nursing home). A) Model for the primary analysis. B) Model for the “screen and treat” analyses. (TIF) [file pone.0035559.s001.tif]

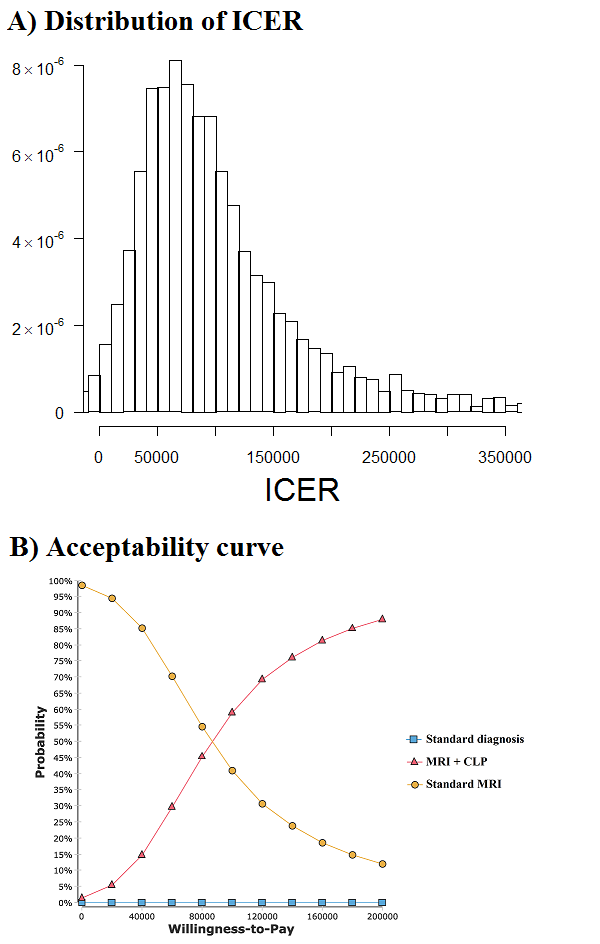

Supplement: Figure S2 — Multivariate sensitivity analysis of the primary cost-effectiveness study. A) Distribution of incremental cost-effectiveness ratios (ICER) of the MRI+CLP strategy, compared to the standard diagnosis strategy. B) Acceptability curve: probability that each strategy either is dominant or has an ICER inferior to the willingness-to-pay, as a function of the willingness-to-pay threshold. (TIF) [file pone.0035559.s002.tif]

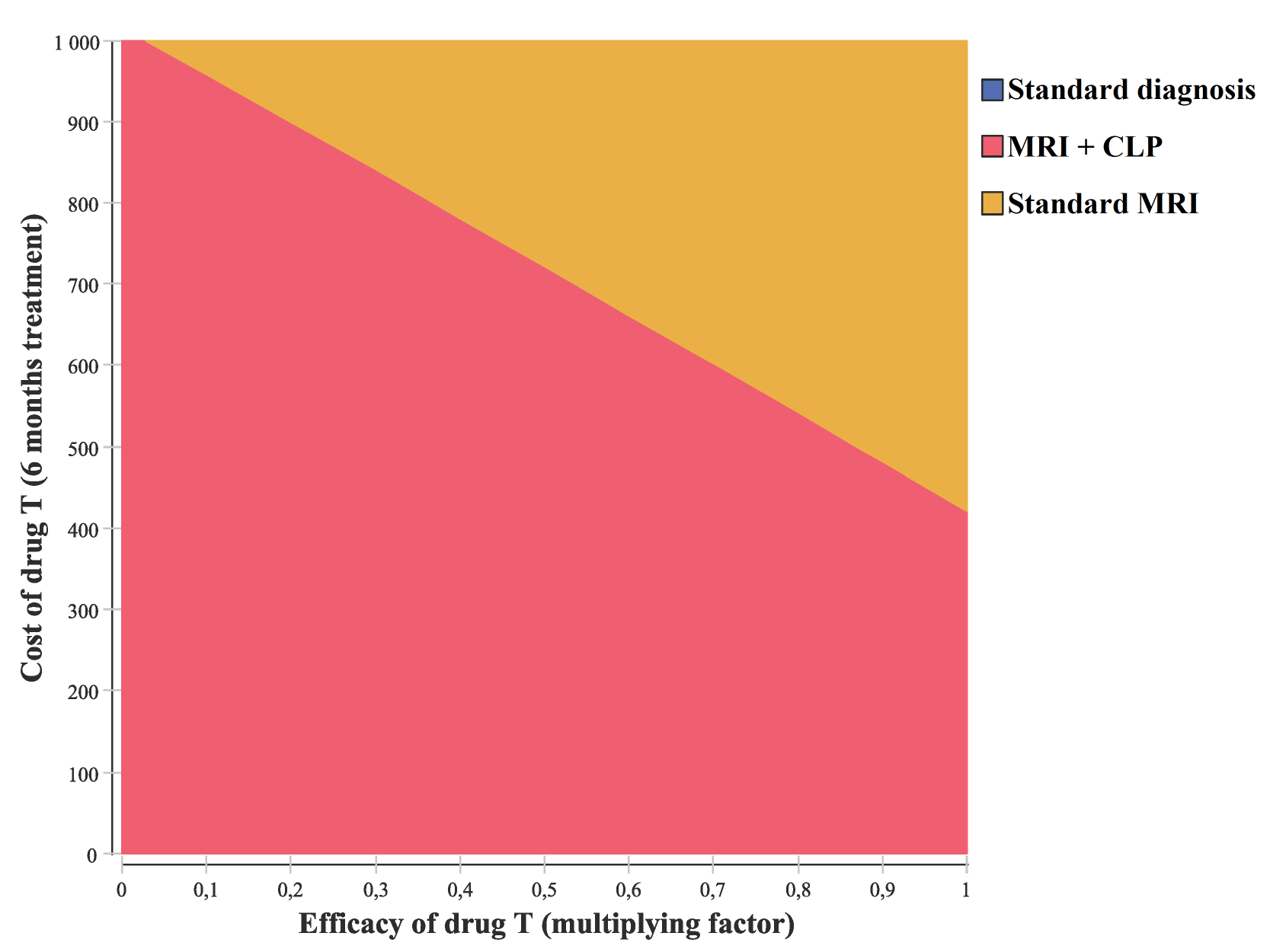

Supplement: Figure S3 — Multivariate sensitivity analysis of the primary cost-effectiveness study with treatment T. The strategy with maximum net monetary benefit is depicted as a function of the assumed efficacy and cost of the hypothetical new drug T. The efficacy of treatment T is expressed as a 0-to-1 ratio between assumed probabilities of transition from early stage AD with and without treatment T; 0 corresponds to maximum efficacy and 1 to no efficacy (in the base case, fT = 0.5: 50% reduction). (TIF) [file pone.0035559.s003.tif]

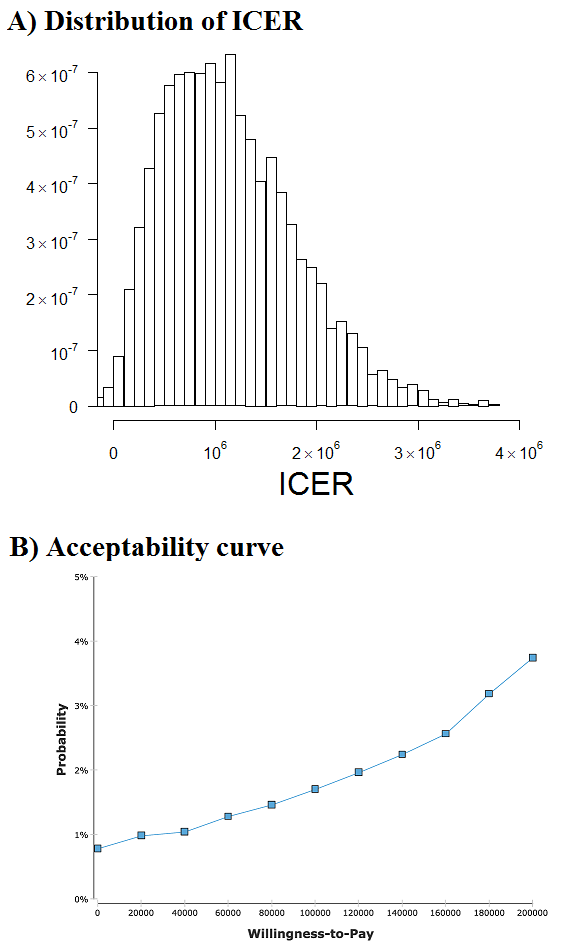

Supplement: Figure S4 — Multivariate sensitivity analysis of the “screen and treat” (population-wide screening) cost-effectiveness study. A) Distribution of incremental cost-effectiveness ratios (ICER) of the MRI+CLP strategy, compared to the standard MRI strategy. B) Acceptability curve: probability that the MRI+CLP strategy either is dominant or has an ICER inferior to the willingness-to-pay, as a function of the willingness-to-pay threshold. (TIF) [file pone.0035559.s004.tif]

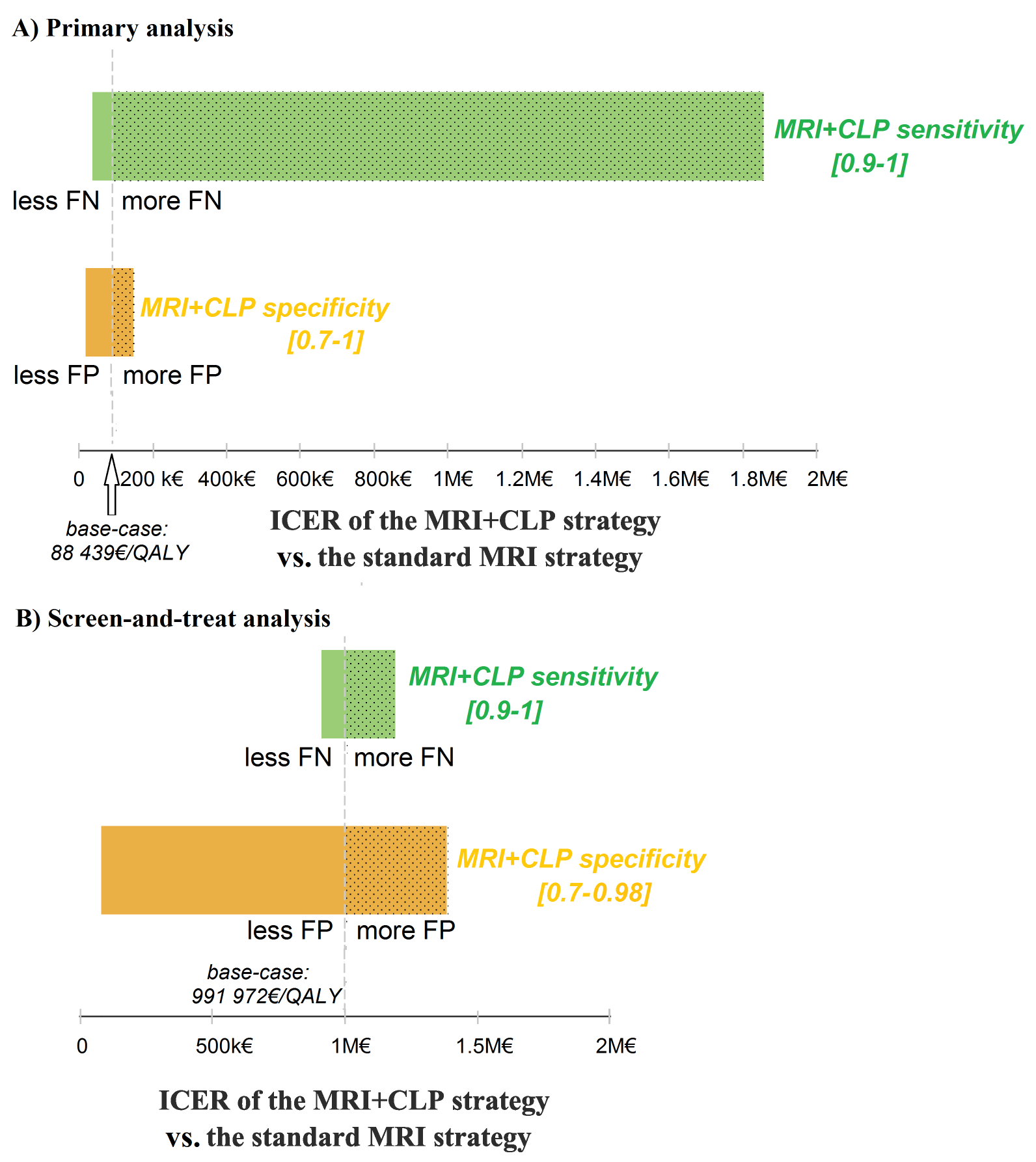

Supplement: Figure S5 — Analysis of the influence of MRI+CLP false positives and false negatives on the cost-effectiveness of the MRI+CLP strategies (tornado diagram). The range of incremental cost-effectiveness ratios (ICER) of the MRI+CLP strategy (compared to the standard MRI strategy) in A) the primary analysis and B) the “screen and treat” analysis is depicted for MRI+CLP sensitivities ranging from 0.9 to 1 and specificities ranging from 0.7 to 1. A sensitivity lower than 0.96 (base-case value) implies a higher risk of false negatives (FN, dotted green bars) than in the base-case. A specificity lower than 0.87 (base-case value) implies a higher risk of false positives (FP, dotted yellow bars) than in the base-case. (TIF) [file pone.0035559.s005.tif]

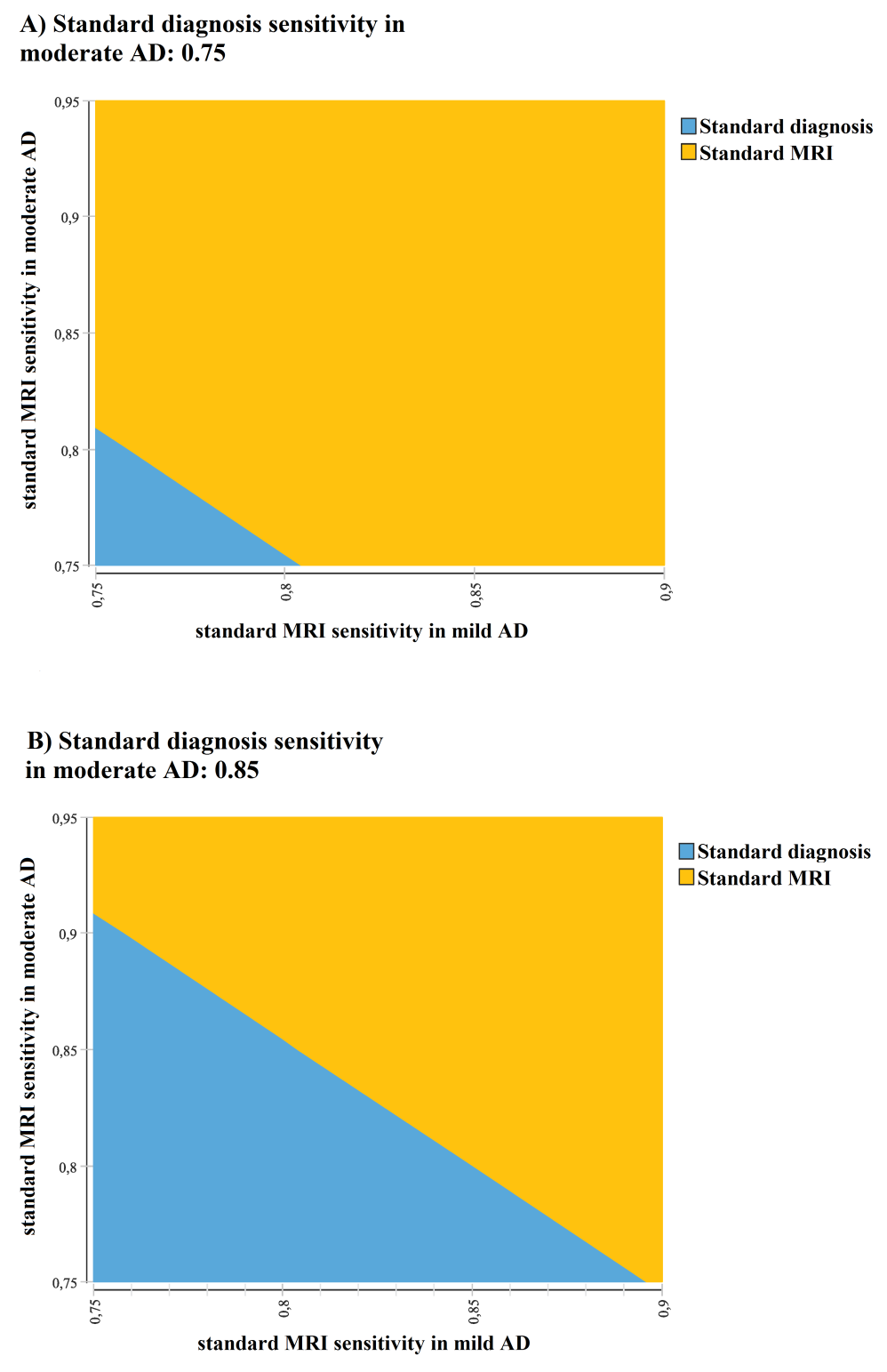

Supplement: Figure S6 — Multivariate sensitivity analysis of the results of the primary cost-effectiveness study on standard MRI vs. standard diagnosis. The strategy with maximum net monetary benefit among these two strategies is depicted as a function of the assumed sensitivity of the standard MRI diagnostic test in mild and moderate AD, for an assumed sensitivity of the standard diagnosis strategy in moderate AD between 0.75 and 0.85 (in the absence of CLP and treatment T). A) 0.75 sensitivity in moderate AD for standard diagnosis (base-case value). B) 0.85 sensitivity in moderate AD for standard diagnosis. (TIF) [file pone.0035559.s006.tif]
