# Supplementary material for: Cost-Effectiveness of Magnetic Resonance Imaging with a New Contrast Agent for the Early Diagnosis of Alzheimer's Disease
Source: PLoS One. 2012 Apr 20;7(4):e35559. doi: 10.1371/journal.pone.0035559 (PMC3332046; doi:10.1371/journal.pone.0035559)
Supplement: File S1 — Models of disease progression. (DOCX) [file pone.0035559.s007.docx]

**File S1 – Models of disease progression**

For both our analyses, we developed state-transition Markov models to characterize the progression of AD through different stages. In the first analysis, in any time period, patients were classified into one of five disease stages: no AD, mild, moderate, or severe AD, or dead. In the second analysis, we added a sixth disease stage for early AD. In both analyses, all living stages were further subdivided in two, for home-cared and institutionalized patients.

All diagnosed patients were assumed to receive treatment; all patients with severe stage AD were assumed to be diagnosed. Patients with mild or moderate AD received a donepezil-like treatment, which was changed to memantine as they progressed to severe AD. Patients diagnosed with severe AD received a memantine-like treatment. In order to distinguish patients with severe AD receiving donepezil from those receiving memantine, the severe stage was separated into two stages: the “severe 1” stage, to which patients already diagnosed with AD first progressed; and the “severe 2” stage, for other severe AD patients. No one could stay in the “severe 1” stage for more than one 6-month cycle.

The models started with an initial distribution of patients in assigned states and applied transition rates recursively over time to simulate how patients progressed through disease stages. In each 6-month cycle, patients faced several possible transitions among various disease stages. For example, a patient initially home-cared and in the mild AD state could remain in that state or progress into one of a number of alternative states (e.g., moderate AD and home-cared or severe 1 AD and home-cared or mild AD and institutionalized). Possible transitions are provided on Supplementary Figure 1, which depicts the models.

Transition rates, which were modified if patients received treatment, are provided in Table 2. As the cohorts cycled through the models, they accrued costs and QALY weights associated with each stage and taking into account the setting (home or institutional care).
