# Supplementary material for: Cost-Effectiveness of Magnetic Resonance Imaging with a New Contrast Agent for the Early Diagnosis of Alzheimer's Disease
Source: PLoS One. 2012 Apr 20;7(4):e35559. doi: 10.1371/journal.pone.0035559 (PMC3332046; doi:10.1371/journal.pone.0035559)
Supplement: Table S1 — Model parameters: base-case values and ranges investigated in the sensitivity analyses. (DOCX) [file pone.0035559.s008.docx]

**Supplementary Table S1 – Model parameters: base-case values and ranges investigated in the sensitivity analyses**

| **Parameter** | | **Value** | | | **Range** | **Source** |
| --- | --- | --- | --- | --- | --- | --- |
| **Primary model transition rates (/6 month)** | | |  | |  |  |
|  | No AD to no AD | 0.939 | | | *computed* | *computed* |
|  | No AD to mild AD | 0.03 | | | +/-10% | [17,26,28] |
|  | No AD to moderate AD | 0.02 | | | +/-10% | [17,26,28] |
|  | No AD to severe AD | 0 | | | +/-10% | [17,26,28] |
|  | No AD to dead | 0.009 | | | *computed* | [20] |
|  |  | ***No treatment*** | ***Under treatment*** | |  |  |
|  | Mild to mild AD | 0.780 | 0.878 | | *computed* | *computed* |
|  | Mild to moderate AD | 0.211 | 0.094 | | +/-10% | [17,21,22,23] |
|  | Mild to severe AD | 0 | 0.019 | | +/-10% | [17] |
|  | Mild AD to dead | 0.009 | 0.009 | | *computed* | [17,20] |
|  | Moderate to mild AD | 0.028 | 0.060 | | *computed* | [17,21,22,23] |
|  | Moderate to moderate AD | 0.744 | 0.813 | | *computed* | [17] |
|  | Moderate to severe AD | 0.207 | 0.103 | | +/-10% | [17,21,22,23] |
|  | Moderate AD to dead | 0.021 | 0.024 | | *computed* | [17,20] |
|  | Severe to moderate AD | - | 0.100 | | *computed* | [24] |
|  | Severe to severe AD | - | 0.820 | | *computed* | *computed* |
|  | Severe AD to dead | - | 0.080 | | +/-10% | [17,20] |
| **“Screen and treat” model transition rates (/6 month)** | | |  | |  |  |
| *At age a: IAD(a)= yearly AD incidence, MR(a)= mortality rate* | | | | |  |  |
|  | No AD to no AD | *Computed* | | | *computed* | *computed* |
|  | No AD to early AD | $0.4\times\left( 1-\sqrt{1-I_{AD}(a)} \right)$ | | | +/-10% | ** |
|  | No AD to mild AD | $0.34\times\left( 1-\sqrt{1-I_{AD}(a)} \right)$ | | | +/-10% | [4,17] |
|  | No AD to moderate AD | $0.24\times\left( 1-\sqrt{1-I_{AD}(a)} \right)$ | | | +/-10% | [4,17] |
|  | No AD to severe AD | $0.02\times\left( 1-\sqrt{1-I_{AD}(a)} \right)$ | | | +/-10% | [4,17] |
|  | No AD to dead | $1-\sqrt{1-MR(a)}$ | | | *computed* | [20] |
|  |  | ***No treatment*** | | ***Under treatment*** |  |  |
|  | Early to early AD | *computed* | | *computed* | *computed* | *computed* ** |
|  | Early to mild AD | 0.026 | | 0.013 | +/-10% | ** |
|  | Early to moderate AD | 0.016 | | 0.008 | +/-10% | ** |
|  | Early to severe AD | 0 | | 0 | +/-10% | ** |
|  | Early AD to dead | $1-\sqrt{1-MR(a)}$ | | $1-\sqrt{1-MR(a)}$ | *computed* | [20] |
|  | Mild to mild AD | *computed* | | *computed* | *computed* | *computed* |
|  | Mild to moderate AD | 0.211 | | 0.094 | +/-10% | [17,21,22,23] |
|  | Mild to severe AD | 0 | | 0.019 | +/-10% | [17] |
|  | Mild AD to dead | $1-\sqrt{1-MR(a)}$ | | $1-\sqrt{1-MR(a)}$ | *computed* | [17,20] |
|  | Moderate to mild AD | 0.028 | | 0.06 | *computed* | [17,21,22,23] |
|  | Moderate to moderate AD | 0.744 | | 0.813 | *computed* | *computed* |
|  | Moderate to severe AD | 0.207 | | 0.103 | +/-10% | [17,21,22,23] |
|  | Moderate AD to dead | 0.021 | | 0.024 | *computed* | [17,20] |
|  | Severe to moderate AD | - | | 0.1 | *computed* | [24] |
|  | Severe to severe AD | - | | 0.820 | *computed* | *computed* |
|  | Severe AD to dead | - | | 0.08 | +/-10% | [17,20] |
| **Transitions from home-care to institutional care (/6 month) in individuals diagnosed as AD patients:** | |  | |  |  |  |
|  | No or early AD | 0.009 | | |  | [19] |
|  | Mild AD | 0.0192 | | |  | [17] |
|  | Moderate AD | 0.0566 | | |  | [17] |
|  | Severe AD | 0.1392 | | |  | [17] |
| **Transitions from home-care to institutional care (/6 month) in undiagnosed individuals:** | |  | | |  |  |
| No or early AD | | 0.009 over 70, 0 under 70 | | |  | [19] |
| Mild AD | | 0.0111 | | |  | [19] |
| Moderate AD | | 0.0187 | | |  | [19] |
| **Prevalence at diagnosis (1^st^ analysis), including:** | | **0.56** | | | **[0.50-0.70]** | **[10]** |
|  | Mild AD | 55.9% | | | [50%-75%] | [27] |
|  | Moderate AD | 39.9% | | | (10/11)×(1-P_mild AD_) | [27] |
|  | Severe AD | 4.2% | | | (1/11)×(1-P_mild AD_) | [27] |
| **Prevalence in the screened population (2^nd^ analysis), including:** | | **0.01** | | | **[0.01-0.10]** | **[3]** |
|  | Early AD | 40% | | | [30%-75%] | ** |
|  | Mild AD | 35% | | | 55.9%×(1-P_early AD_) | [27] ** |
|  | Moderate AD | 21% | | | 39.9%×(1-P_early AD_) | [27] ** |
|  | Severe AD | 4% | | | 4.2%×(1-P_early AD_) | [27] ** |
| **Sensitivity in early AD** | |  |  | |  |  |
|  | Standard diagnosis | 0.10 | | |  | ** |
|  | Standard MRI | 0.50 | | | [0-0.5] | ** |
|  | MRI + CLP | 0.96 | | | [0.90-1] | [32,33] |
| **Sensitivity in mild AD** | |  |  | |  |  |
|  | Standard diagnosis | 0.75 | | |  | [10] |
|  | Standard MRI | 0.88 | | | [0.75-0.90] | [31] |
|  | MRI + CLP | 0.96 | | | [0.90-1] | [32,33] |
| **Sensitivity in moderate AD** | |  |  | |  |  |
|  | Standard diagnosis | 0.75 | | | [0.75-0.90] | [10] |
|  | Standard MRI | 0.95 | | | [0.75-0.95] | [31] |
|  | MRI + CLP | 0.96 | | | [0.90-1] | [32,33] |
| **Specificity** | |  |  | |  |  |
|  | Standard diagnosis | 0.90 | | | [0.75-0.90] | [10] |
|  | Standard MRI | 0.96 | | | [0.75-0.96] | [31] |
|  | MRI + CLP | 0.87 | | | [0.70-1] | [32,33] |
| **Costs (in 2009 €, for 6 months)** | |  |  | |  |  |
| Diagnosis | |  |  | |  |  |
|  | 1^st^ consultation | 55 | | |  | [34] |
|  | Follow-up visits | 41 | | |  | [34] |
|  | Laboratory tests | 50 | | |  | [36] |
|  | MRI equipment | 282 | | |  | [35] |
|  | MRI procedure | 69 | | |  | [35] |
|  | Classical contrast agent | 5 | | |  | [35] |
|  | CLP contrast agent | 250 | | | [0-500] | *Assumed* ^*^ |
|  | Contrast agent injection | 10 | | |  | [35] |
|  | MMSE test | 69 | | |  | [35] |
| Treatment | |  |  | |  |  |
|  | Donepezil treatment | 572.4 | | |  | [37] |
|  | Memantine treatment | 286.2 | | |  | [37] |
|  | T treatment | 500 | | | [0-1000] | *Assumed* |
| Basic caregiving and living costs | |  |  | |  |  |
|  | No or early AD, home care | 2001.6 | | |  | [39] |
|  | No or early AD, institution | 1825.20 | | |  | [18] |
|  | Mild AD, home care | 3151.20 | | |  | [18,38,39] |
|  | Mild AD, institution | 4719 | | |  | [18,38] |
|  | Moderate AD, home care | 7934.10 | | |  | [18,38,39] |
|  | Moderate AD, institution | 6643 | | |  | [18,38] |
|  | Severe AD, home care | 26630,4 | | |  | [18,38,39] |
|  | Severe AD, institution | 8664 | | |  | [18,38] |
| Indirect costs | |  |  | |  |  |
|  | Informal volunteer activities | 344 | | |  | [41,42] |
|  | NPO volunteer activities | 199 | | |  | [41,43] |
|  | Efficiency coefficient | 0.7 | | |  | *Assumed* |
|  | Resulting productivity benefit, moderate-to-severe AD | 543 | | |  | *Computed* |
|  | Resulting productivity benefit, mild AD | 326 | | |  | *Computed* |
|  | Health impact on caregivers | 569 | | |  | [34,37,38,40] |
| **QALYs** |  |  |  | |  |  |
|  | No AD (65-84 y.o.) | 0.826 | | |  | [44] |
|  | Early AD | 0.826 | | |  | [44] |
|  | Mild AD, home care | 0.68 | | |  | [17] |
|  | Mild AD, institution | 0.71 | | |  | [17] |
|  | Moderate AD, home care | 0.54 | | |  | [17] |
|  | Moderate AD, institution | 0.48 | | |  | [17] |
|  | Severe AD, home care | 0.37 | | |  | [17] |
|  | Severe AD, institution | 0.31 | | |  | [17] |
| **Discount rate (/ year)** | | 5% | | | [0-10%] | *Assumed* |

^*^ Personal communication, Guerbet company, 2009

** Personal communication, Pr. M. Verny, AP-HP, 2009
