# Supplementary material for: Cost-Effectiveness of Magnetic Resonance Imaging with a New Contrast Agent for the Early Diagnosis of Alzheimer's Disease
Source: PLoS One. 2012 Apr 20;7(4):e35559. doi: 10.1371/journal.pone.0035559 (PMC3332046; doi:10.1371/journal.pone.0035559)
Supplement: Table S2 — Univariate sensitivity analysis: incremental cost-effectiveness ratio (ICER) of the MRI+CLP strategy, compared to the reference strategy, in the primary cost-effectiveness analysis, depending on the values of model parameters. (DOCX) [file pone.0035559.s009.docx]

**Supplementary Table S2 – Univariate sensitivity analysis: incremental cost-effectiveness ratio (ICER) of the MRI+CLP strategy, compared to the reference strategy, in the primary cost-effectiveness analysis, depending on the values of model parameters.**

| **Model parameter** | **ICER of the MRI+CLP strategy (€/QALYs)** | **Preferred strategy** |
| --- | --- | --- |
| Drug efficacy f ^*^ |  |  |
| 0.5 | **22 117 (<WTP)** | MRI+CLP |
| 0.6 | **30 088 (<WTP)** | MRI+CLP |
| 0.7 | **39 920 (<WTP)** | MRI+CLP |
| 0.8 | **52 116 (<WTP)** | MRI+CLP |
| 0.9 | **67 797 (<WTP)** | MRI+CLP |
| 1 | 88 439 | Standard MRI |
| MRI+CLP sensitivity |  |  |
| 0.9 | 1 778 385 | Standard MRI |
| 0.92 | 305 676 | Standard MRI |
| 0.94 | 147 955 | Standard MRI |
| 0.96 | 88 439 | Standard MRI |
| 0.98 | **57 039 (<WTP)** | MRI+CLP |
| 1 | **37 569 (<WTP)** | MRI+CLP |
| MRI+CLP specificity |  |  |
| 0.70 | 146 876 | Standard MRI |
| 0.75 | 134 997 | Standard MRI |
| 0.80 | 115 862 | Standard MRI |
| 0.84 | 100 967 | Standard MRI |
| 0.88 | 83 741 | Standard MRI |
| 0.92 | **64 689 (<WTP)** | MRI+CLP |
| 0.96 | **42 838 (<WTP)** | MRI+CLP |
| 1 | **17 524 (<WTP)** | MRI+CLP |
| AD progression speed |  |  |
| -10% | 113 399 | Standard MRI |
| -6% | 102 878 | Standard MRI |
| -2% | 93 209 | Standard MRI |
| +0% | 88 349 | Standard MRI |
| +2% | 83 903 | Standard MRI |
| +6% | **75 739** **(<WTP)** | MRI+CLP |
| +10% | **67 891 (<WTP)** | MRI+CLP |
| Initial AD prevalence |  |  |
| 0.5 | 105 347 | Standard MRI |
| 0.55 | 91 136 | Standard MRI |
| 0.6 | 78 512 | Standard MRI |
| 0.65 | **66 927 (<WTP)** | MRI+CLP |
| 0.7 | **56 828 (<WTP)** | MRI+CLP |
| Initial portion of mild stage AD patients | |  |
| 0.5 | 97 175 | Standard MRI |
| 0.55 | 89 682 | Standard MRI |
| 0.6 | 83 541 | Standard MRI |
| 0.65 | 77 757 | Standard MRI |
| 0.7 | **72 637 (<WTP)** | MRI+CLP |
| 0.75 | **68 073 (<WTP)** | MRI+CLP |

^*^ assuming that $\text{f}\text{=}\text{f}_{\text{m}\text{M}}\text{=}\text{f}_{MS}$ and that $\text{f}_{\text{M}\text{m}}\text{=}\text{f}_{\text{SM}}\text{=-2×}\text{f}\text{+3}$

**Supplementary Table S2 (continued)**

| Cost of the CLP contrast agent | |  |
| --- | --- | --- |
| 0 | **28 918 (<WTP)** | MRI+CLP |
| 50 | **40 782 (<WTP)** | MRI+CLP |
| 100 | **52 645 (<WTP)** | MRI+CLP |
| 250 | 88 439 | Standard MRI |
| 500 | 147 553 | Standard MRI |
| Discount rate for costs and QALYs | |  |
| 0 | 78 336 | Standard MRI |
| 2% | 82 390 | Standard MRI |
| 4% | 86 357 | Standard MRI |
| 6% | 90 175 | Standard MRI |
| 8% | 94 743 | Standard MRI |
| 10% | 98 636 | Standard MRI |
| Sensitivity of the standard diagnosis in mild stage AD | |  |
| 0.75 | 88 439 | Standard MRI |
| 0.80 | 88 439 | Standard MRI |
| 0.85 | 88 439 | Standard MRI |
| 0.90 | 88 439 | Standard MRI |
