# Supplementary material for: Cost-Effectiveness of Magnetic Resonance Imaging with a New Contrast Agent for the Early Diagnosis of Alzheimer's Disease
Source: PLoS One. 2012 Apr 20;7(4):e35559. doi: 10.1371/journal.pone.0035559 (PMC3332046; doi:10.1371/journal.pone.0035559)
Supplement: Table S3 — Univariate sensitivity analysis: ICER of the MRI+CLP strategy compared with the reference strategy, in the universal “screen and treat” cost-effectiveness analysis, depending on the values of model parameters. (DOCX) [file pone.0035559.s010.docx]

**Supplementary Table S3 – Univariate sensitivity analysis: ICER of the MRI+CLP strategy compared with the reference strategy, in the universal “screen and treat” cost-effectiveness analysis, depending on the values of model parameters.**

| **Model parameter** | **ICER of the MRI+CLP strategy (€/QALYs)** | | **Preferred strategy** |
| --- | --- | --- | --- |
| Efficacy of current drugs f ^*^ | | |  |
| 0.5 | 1 025 885 | | Standard MRI |
| 0.6 | 1 020 102 | | Standard MRI |
| 0.7 | 1 009 113 | | Standard MRI |
| 0.8 | 1 008 768 | | Standard MRI |
| 0.9 | 998 047 | | Standard MRI |
| 1 | 991 973 | | Standard MRI |
| Efficacy of drug T f_T_ | | |  |
| 0.05 | | 557 316 | Standard MRI |
| 0.25 | | 707 978 | Standard MRI |
| 0.5 | | 991 973 | Standard MRI |
| 0.75 | | 1 504 799 | Standard MRI |
| 0.95 | | 2 374 250 | Standard MRI |
| Cost of drug T | |  |  |
| 0 | | 311 447 | Standard diagnosis |
| 250 | | 639 606 | Standard MRI |
| 500 | | 991 973 | Standard MRI |
| 750 | | 1 345 651 | Standard MRI |
| 1000 | | 1 698 673 | Standard MRI |
| MRI+CLP sensitivity |  | |  |
| 0.9 | 1 115 013 | | Standard MRI |
| 0.92 | 1 071 041 | | Standard MRI |
| 0.94 | 1 030 365 | | Standard MRI |
| 0.96 | 991 973 | | Standard MRI |
| 0.98 | 957 525 | | Standard MRI |
| 1 | 924 790 | | Standard MRI |
| MRI+CLP specificity |  | |  |
| 0.7 | 1 380 332 | | Standard MRI |
| 0.85 | 1 065 093 | | Standard MRI |
| 0.90 | 844 965 | | Standard MRI |
| 0.95 | 461 603 | | Standard MRI |
| 0.96 | 350 277 | | Standard MRI |
| 0.97 | 223 721 | | Standard MRI |
| 0.98 | **73 000 (<WTP)** | | MRI+CLP |
| 0.99 | **Dominant** | | MRI+CLP |
| 1 | **Dominant** | | MRI+CLP |
| Standard MRI sensitivity in early AD |  | |  |
| 0.1 | 602 140 | | Standard MRI |
| 0.3 | 761 117 | | Standard MRI |
| 0.5 | 991 973 | | Standard MRI |

^*^ assuming that $\text{f}\text{=}\text{f}_{\text{mM}}\text{=}\text{f}_{MS}$ and that $\text{f}_{\text{M}\text{m}}\text{=}\text{f}_{\text{SM}}\text{=-2×}\text{f}\text{+3}$

**Supplementary Table S3 (continued)**

| **Model parameter** | **ICER of the MRI+CLP strategy (€/QALYs)** | **Preferred strategy** |
| --- | --- | --- |
| AD progression speed |  |  |
| -10% | 1 143 465 | Standard MRI |
| -5% | 1 059 971 | Standard MRI |
| +0% | 991 973 | Standard MRI |
| +5% | 928 622 | Standard MRI |
| +10% | 872 164 | Standard MRI |
| Cost of the CLP contrast agent | |  |
| 0 | 779 307 | Standard MRI |
| 250 | 991 973 | Standard MRI |
| 500 | 1 205 950 | Standard MRI |
| 750 | 1 419 272 | Standard MRI |
| 1000 | 1 632 593 | Standard MRI |

| Initial AD prevalence |  |  |
| --- | --- | --- |
| 0.01 | 991 973 | Standard MRI |
| 0.03 | 495 113 | Standard MRI |
| 0.05 | 321 561 | Standard MRI |
| 0.06 | 271 231 | Standard MRI |
| 0.08 | 203 600 | Standard MRI |
| 0.1 | 160 246 | Standard MRI |
| Initial portion of early stage AD patients | |  |
| 0.3 | 1 114 662 | Standard MRI |
| 0.4 | 991 973 | Standard MRI |
| 0.5 | 890 202 | Standard MRI |
| 0.6 | 810 022 | Standard MRI |
| 0.7 | 739 986 | Standard MRI |
| 0.75 | 709 233 | Standard MRI |
| Discount rate |  |  |
| 0% | 866 323 | Standard MRI |
| 2% | 911 122 | Standard MRI |
| 4% | 961 076 | Standard MRI |
| 6% | 1 016 343 | Standard MRI |
| 8% | 1 082 440 | Standard MRI |
| 10% | 1 147 824 | Standard MRI |
| Screening frequency |  |  |
| Every year | 2 866 413 | Standard MRI |
| Every 5 years | 991 973 | Standard MRI |
| Every 10 years | 307 117 | Standard MRI |
